# Mission-oriented innovation for sustainable polymers in liquid formulation

**DOI:** 10.1098/rsta.2023.0272

**Published:** 2024-09-23

**Authors:** Anju Massey-Brooker, Rowan Conway

**Affiliations:** ^1^ Department of Chemistry, Durham University, Stockton, UK; ^2^ UCL Institute of Innovation and Public Purpose, London, UK; ^3^ Just Transitions Finance Lab, Grantham Institute for Climate Change and the Environment, London School of Economics, London, UK

**Keywords:** polymers in liquid formulation, missions, sustainability

## Abstract

Industrial chemical producers and formulators are increasingly conscious of their responsibility in stewarding planetary resources and minimizing harm to the environment. In 2019, the Royal Society of Chemistry (RSC) engaged an industry task force from across the value chain to drive technical research to classify a new class of polymer—polymers in liquid formulation (PLFs). Building on this, the task force called for step change in sustainability practices for PLFs and instigated a design and development process to identify research themes and priorities that could accelerate innovation in this area. However, a key challenge was that as a novel classification, PLFs were largely unknown outside the chemistry community and entirely absent from the mainstream research agenda. To establish the demand-pull requirements of the value chain for sustainable PLFs, the RSC used a ‘mission-oriented’ innovation framework to enable the taskforce to co-design an ideal-type portfolio of research and innovation projects, and to set out a realistic roadmap for transition. This perspective article presents a summary of the activities carried out by the task force in its pursuit of mission-oriented innovation for PLFs and describes the strategic design method used to enable cross-value chain consensus on action for PLF sustainability, build system-wide innovation ecosystems and explore common-good scenarios.

This article is part of the discussion meeting issue ‘Green carbon for the chemical industry of the future’.

## 1. Introduction

Industrial chemical producers and formulators are increasingly conscious of their responsibility in stewarding planetary resources and minimizing harm to the environment [1], and yet the chemicals industry lags behind others in terms of sustainability transitions [2]. With net-zero transition pressure growing across industries [3] chemical producers and formulators are becoming increasingly conscious of their responsibility in stewarding resource use and minimizing harm to the environment [4,5]. This article looks at the case of polymers in liquid formulation (PLFs), and describes a process driven by the Royal Society of Chemistry (RSC) to first classify this new class of polymer through technical research, and then to identify the demand-side innovation requirements and set out a roadmap to sustainability using an applied mission-oriented innovation design process.

PLFs are a high-value, a critically important class of speciality chemicals [6] that can be grouped into nine key types of materials: acrylic, epoxy resins, polyesters, polysilicones, polyurethanes, radiation-curable, vinyl, water-soluble and other low-volume polymers [7]. PLF products are either liquid formulation systems that remain as a liquid in use (such as personal care products) or curable formulation systems that form solids on application (such as adhesives or sealants). Owing to the exceptional complexity of this diverse class of material and its wide variety of uses, the environmental effects of PLFs in terms of carbon release, resource usage and waste generation cannot easily be quantified, but the RSC estimates that the global production volume for PLFs is 29 million tonnes per annum [7], representing a significant sustainability challenge to mitigate environmental harm and lessen fossil-fuel dependence.

To define a vision for sustainable PLFs, the RSC engaged an industry task force from across the value chain. The PLF value chain is a complex one, and the RSC’s Sustainable PLF Task Force reflected this complexity [8]. It was comprised of high-level representation from Afton Chemical, BASF, Croda, Crown Paints, Dow, Northumbrian Water, Scott Bader, Unilever, United Utilities and Walgreens Boots Alliance and its remit was to establish a clear industry-wide sustainability agenda for PLFs. The work began with a technical report on PLFs and then followed a deliberative design process to set out missions and build a roadmap to shift the production, use and disposal practices of incumbent PLF towards a sustainable path—a challenge that was seen as too large for any single firm to address alone.

## 2. Sustainable PLF: the technical foundations

**Figure 1.**
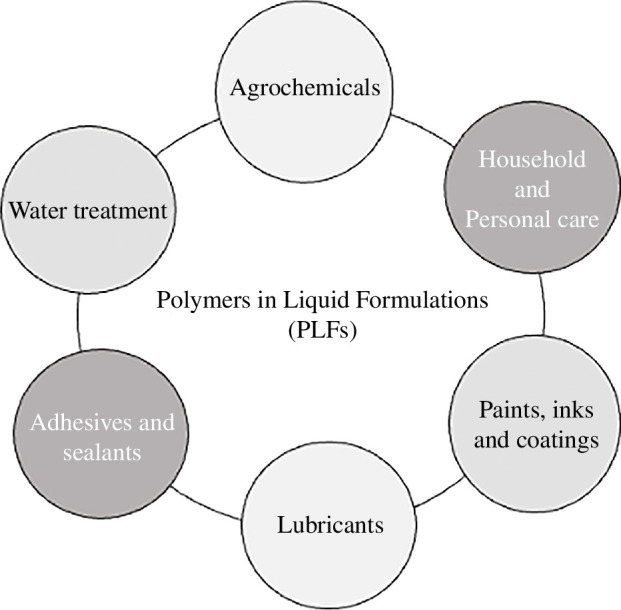
PLF industries.

In 2019, the sustainability challenges presented by PLFs were detailed in a comprehensive technical report. PLFs are present in six key industries: agrochemicals, household and personal care, paints and coatings, water treatment, adhesives and sealants and lubricants (see figure 1). Despite their importance to society and the global economy, there has been very little coordinated effort to highlight the sustainability problems surrounding them. The RSC aimed to address this gap and the first stage of the PLF programme was to produce a technical report to provide a coherent knowledge base.

The sustainability challenges of PLFs are vast but not visible. While plastics have received widespread attention for their negative environmental effect on biodiversity and marine ecosystems [9], by contrast, PLFs are largely unknown outside the chemistry community. And yet they are commonly used as thickeners, emulsifiers and binders in household cleaning and personal care, and in a range of industrial applications including agriculture and wastewater treatment.

Broader sustainability pressures on industrial producers and formulators have illuminated the need to understand the problems associated with PLFs. Consumer pressure also plays a role, and formulators are facing demand for greener products. Across the value chain, firms are cognisant of impending regulatory pressures when the European Commission amends its REACH regulations in the near future [10].

Preparedness is key: industrial systems need certainty if they are to invest in infrastructure or create large-scale changes to supply chains. The sustainability challenges of PLFs are varied. RSC research shows that 36 million tonnes of PLFs are made and sold each year, many of which are produced in sub-gram quantities, registered for production in a variety of ways and produced as single entities or combination materials [7]. Resource security is a notable problem across the value chain, from monomer producers to product formulators and waste management companies.

While they are ubiquitous, PLFs are difficult to monitor and connect directly to targets for reduction of greenhouse gas emissions. This means that they do not directly align with corporate sustainability targets or government priorities. What is known is that the production, use and storage of PLFs puts a significant strain on the environment—releasing carbon, using finite resources and generating waste and pollution.

## 3. Going beyond net zero

While net zero dominates the political and corporate discourse, sustainability is a multifaceted challenge in the chemicals sector and includes a range of environmental pressures, from feedstock extraction to end-of-life waste and pollution. The RSC’s technical report into PLFs highlighted three key pressures:

— Reliance on fossil-derived feedstocks as raw materials for producing PLF products.
— Waste production from PLF products that are disposed of at the end of their life.
— Mitigating the volume of pollution entering the environment from PLFs.

In 2023, the RSC undertook stakeholder research with industry, government and academia, which showed that a narrow focus on the connection between PLFs and net zero would not suffice for the broad challenges of PLFs. It became clear that the planetary-boundaries framework would have greater salience. Planetary boundaries are a set of nine quantitative environmental thresholds devised by the Stockholm Institute [11]. These are climate change, ocean acidification, ozone-layer depletion, air pollution, biodiversity loss, land conversion, freshwater withdrawals, nitrogen and phosphorus loading and chemical pollution.

Stockholm Institute research shows that key boundaries in novel entities (which includes plastic and other man-made chemicals) and freshwater were transgressed in 2022 and states that current increasing trends of chemical production and release put the health of the Earth system at risk [12]. At an applied level, some of the descriptors that feature at a high level within the planetary-boundaries framework are also embedded in life-cycle assessment (LCA) impact categories, and so LCA could be an important translation device from the high-level challenges into individual-use cases. For sustainable PLFs, it is also key to quantify the environmental effect of PLFs for different applications (e.g. agrochemistry) and design routes for their manufacture, use, recycling and disposal using cradle-to-grave scenarios.

While the industry is working hard to reduce its carbon footprint, consumer demand for PLFs can and will only increase as the global population grows [13] driving further pressure on planetary boundaries. This in turn will contribute to rises in material production and waste generation, which are already expected to double by 2050 [14]. Within the bio-economy, there are significant opportunities for the development of bio-based building-block chemicals for polymers [15] and industry considers chemicals and polymer production from renewable resources an attractive value proposition [16]. But the fear of high upfront costs and the threat of lower performance versus incumbent petrochemicals pose a significant risk.

## 4. Redressing the 100-year head start

The history of synthetic polymers is deeply tied to fossil fuels. Bio-based polymers have existed for centuries, but petrochemical feedstocks are the default in manufacturing systems owing to over a century of investment in infrastructure, production processes and supply chains. The resulting efficiencies effectively tether production to fossil feedstocks. While it is technically feasible to substitute fossil-derived ingredients with alternative feedstocks [17], the 100-year head start that petrochemicals have had makes the viability of this transition very challenging for industry. To reduce dependence on fossil-derived feedstocks, solutions must be found that are economically viable and available at an industrial scale.

The sustainability discourse is moving beyond ‘carbon tunnel vision’ [18,19], which narrowly focuses on carbon emissions [20] and is beginning to explore the need for defossilization [17,21] across the value chain. A coalition of countries, governments, businesses and scientists have joined the Fossil Fuel Non-Proliferation Treaty Initiative to support the phase-out of fossil-fuel production altogether [22]. And industry is taking its own action to move away from fossil carbons, as seen in the Clean Futures initiative led by Unilever which has defined a Carbon Rainbow—a scheme that categorizes chemical feedstocks in different colours depending on their derivation, the goal of which is to enable an orderly phase out of conventional fossil-fuel derived black carbon by 2030 [23].

However, in innovation systems, Grubb *et al.* [24] have pointed to the embedded inertia in systems of production and consumption that effectively prevent a rapid response to the defossilization challenge. This is commonly attributed to a path-dependent process known as carbon lock-in [19], whereby the conditions that are embedded in incumbent fossil-based industrial systems of production, have locked in the efficiency gains of over a century of industrial development and compounded economic returns to scale. This lock-in acts as a powerful inhibitor of innovation and threatens the competitiveness of low-carbon alternatives [19], as Seto *et al.* [19] note, because ‘innovation is cumulative, multi-faceted and self-reinforcing in its direction’ this path dependency constitutes a perennial head start for the fossil-fuel chemical architecture, making defossilization at industrial scale a complex and expensive task.

The RSC PLF technical report outlined that to transform systems of production and supply for PLFs will require a global policy mandate, large-scale capital allocation and coordinated investment in innovation and infrastructure which currently do not exist. However, this transformation needs to happen in a rapid timeframe with minimal disruption to the consumer experience. This means that solutions must match the performance of existing PLFs across applications, perform in a formulation alongside other ingredients and be sustainable throughout the life cycle. This seemingly impossible task was the starting point for the Sustainable PLF Task Force to begin a mission-oriented innovation process.

## 5. Sustainable PLF: a mission-oriented innovation approach

With PLFs absent from the mainstream research agenda, the RSC used a mission-oriented innovation framework to catalyse a portfolio of collaborative research and innovation [25]. In this section, we look at the co-design process that led to consensus on action for PLF sustainability challenges [26] and demonstrate how this method provided a unique forum to build system-wide innovation ecosystems and explore common-good scenarios. Finding solutions to sustainability challenges is increasingly the driving force for research and innovation [27], and there is a growth in mission-oriented innovation policy led by the European Commission and the EU’s Horizon 2020 funding scheme, which is reorienting innovation towards global grand challenges [28].

**Figure 2.**
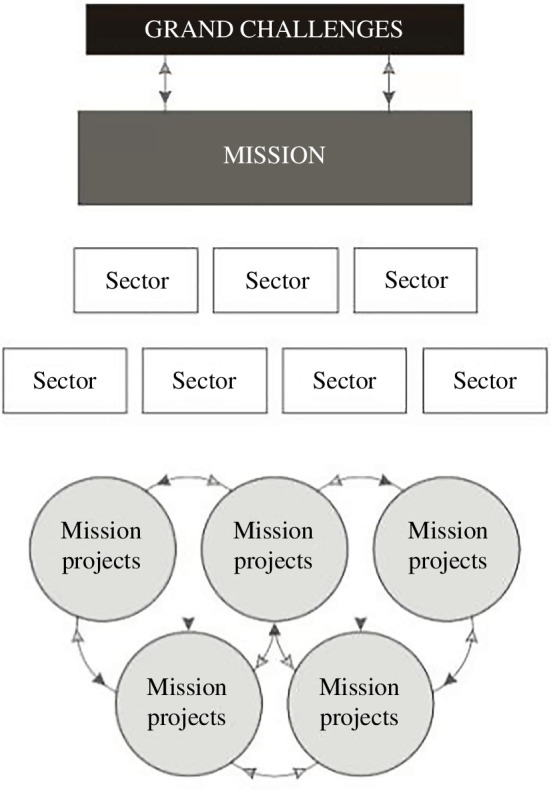
The Mission-Oriented Innovation Framework (Mazzucato, 2021).

To draw industrial production and consumption of PLFs within planetary boundaries will require unprecedented industrial collaboration at scale and the rapid adoption of a range of innovations. The collective challenge is to shift to zero-harm PLFs which are benign by design [29] and coordinate a large-scale shift to sustainable practices. To take on such a task requires a catalytic approach to innovation. The RSC chose to use a mission framework to design a portfolio of collaborative research and innovation because missions offer a lens through which to re-imagine innovation as a vehicle for sustainable development [30]. Popularized by the economist Mariana Mazzucato [30], mission-oriented innovation is generally seen as a top-down policy instrument that provides directionality to a process of bottom-up experimentation [31] (see figure 2, the mission framework developed by Mariana Mazzucato).

In this instance, however, the top-down policy environment did not speak specifically to the problems of PLFs (as they are largely absent from the mainstream research and policy agenda). There are, however, many high-level mandates that do seek to address the associated grand challenge of ‘hazardous chemicals and air, water and soil pollution and contamination’ [32]. The PLF task force had to develop connecting narratives from the bottom up and define tangible missions that could link PLFs to high-level policy mandates such as the sustainable development goals to make these challenges visible to the wider innovation eco-system.

## 6. A process of co-design

Bringing together actors from across the value chain was critical to this task as it illuminated the breadth of the problem and established the demand-pull needs of the whole value chain. This collective purview was crucial, as incremental firm-level innovation is not enough to drive systemic shifts of this scale [33]. The OECD Innovation observatory OPSI says: ‘Short-term, isolated, single stakeholder approaches are no longer sufficient to tackle systemic societal challenges. Mission-oriented innovation policies, governance and practices support directed action towards achieving ambitious goals’ [34]. Missions work to tackle complex challenges by taking a purpose-oriented, market-shaping approach [27] at a scale beyond that which single firms can address alone.

The RSC, as an independent body, provided an important ‘third space’ [35] for industry actors to come together to deliberate on missions and to explore how to ‘de-risk the economic burden of research and development and reduce the potential for duplicating work … [and] overcome technical feasibility and economic challenges of integrating solutions in industry’ [36]. This was a collaborative endeavour in which the task force produced ambitious industry-wide goals, unencumbered by the limitations of single firm constraints—while also considering the technical boundaries set by what was feasible to deliver.

**Figure 3.**
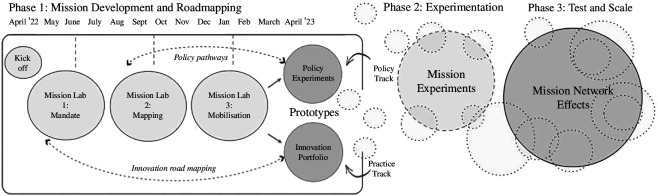
The Mission co-design method (Conway, 2022, unpublished data).

Using a participatory design methodology developed by one of the authors [37] (see figure 3), the group worked through a process of three ‘Mission Labs’ to create high-level goals that could both provide the long-term direction to bring sectors together, while also honing in on practical action and near term priorities. This set out the agenda for decisive action with specific tangible objectives.

Over the course of nine months, the task force worked with facilitators to co-design two missions and set out an innovation roadmap in the Mission Labs. These were half-day workshops held on MS Teams, where the aim was to define a ‘North Star’ [38]—a clear goal for the sustainability transition for the industry—and explore innovation pathways to reach it. Within the lab sessions, small groups co-designed the elements that a transition might need—cataloguing the different types of research, innovations, services and policy mixes that would constitute a portfolio of systems innovation, as well as detailing the requisite roles needed from a range of actors such as policymakers, start-ups or scientists. Through this process, the group developed a common vocabulary and produced a systemic gap analysis that illustrated what would be required to move from the current state to the ideal state, and what incentives would be needed to overcome the inherent barriers to change.

Throughout this design process, the task force played a critical role in the co-production of missions [39] and developed an actionable roadmap by co-creating a speculative future innovation system, and mapping out the resources and business models needed to deliver it. The prototype that they co-designed gave form to a speculative innovation portfolio illustrating the ideal R&I ecosystem that would be required to move beyond the sustainability-as-usual approach [40].

## 7. Defining an overarching sustainability framework

To ensure that the full spectrum of PLF challenges was being addressed through the design process, it was important to also prototype a broader sustainability framework. The second Mission Lab hosted a focused design session to zoom out from the technical detail and define a holistic framework that could speak to the full life cycle—not just the fate end—to avoid issues of narrow silo-creation or burden shifting that can occur in sustainability processes [41]. This prototyping session defined a spectrum of PLF issues in five key challenge areas: Feedstocks, Functionality, Formulation, Fate and Futures, which set out high-level impact categories needed to illustrate the whole life cycle of the PLF.

**Figure 4.**
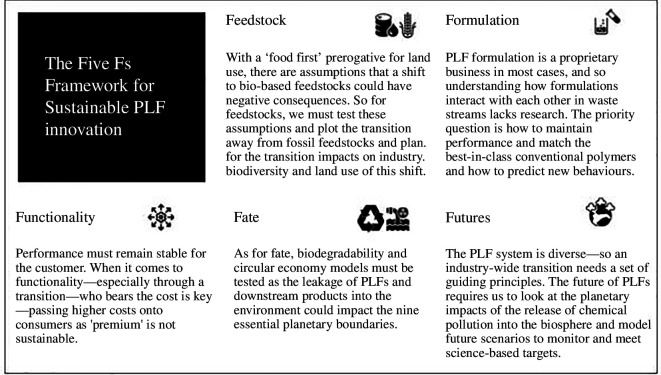
The five Fs framework for sustainable PLF (RSC, 2023).

This framework then acted as an heuristic device to set the boundary space for what could inform the full sustainability discourse covering the cradle-to-grave scenario for PLFs. Defining this framework aimed to support practitioners to focus their efforts on the most relevant effects—while not losing sight of the greater holistic picture. Speculative frames were drafted for discussion to summarize at a high level the sustainability issues for each challenge area. These frames were based on a synthesis of stakeholder interviews and input from the task force members. Their purpose was to give form to some of the issues that currently do not feature on the research and innovation agenda owing to the novelty of PLFs as a research domain. The five Fs Framework is illustrated in figure 4.

## 8. The missions

The five Fs framework provided the big picture of the sustainability challenge for PLFs, but the missions needed to be more concrete and actionable. The missions aim to work on two levels: tackling urgent problems in the short term, while also transforming entire systems of production and consumption towards sustainability in the long term. The near-term challenge of PLFs is end-of-life disposal, and the fate of PLFs is a technically complex challenge that no single actor in the value chain could tackle alone. PLF materials enter the environment at end of life in a variety of ways from solid waste disposal and collection, to release into air, land and marine environments. Different types of waste will accompany each use, for example, paints and coatings will vary from crop protection or personal care.

Through the Mission Labs, the task force decided on two missions. The first was to ‘Develop and Scale Biodegradable PLF by 2030’. This biodegradability focus was deemed essential, especially for those polymers that are washed away into drains and are hard to recover from household care, personal care and cosmetics. It also revealed an actionable challenge in that the OECD standards set for biodegradability did not track directly to PLFs— presenting a tangible entry point for action.

The second mission was to ‘Advance circular economy infrastructure for PLFs by 2030’ [23]. This mission sought to connect PLFs to a growing policy agenda for transitioning to a circular economy [42], and participants explored how to move from virgin fossil-derived feedstocks towards green carbon sources. While there is an abundance of high-level policy direction for a circular economy, practical detail is less readily available, and it is as yet unclear what circular infrastructure for PLFs might look like. There is also no incentive for single firms to invest in costly circular infrastructure, in that it essentially delivers industry-wide benefit but may entail a first mover disadvantage.

This lack of incentive has acted as an inhibitor to progress, so adding the word ‘advance’ to the mission was important as it emphasizes progress, not perfection. It also does not overly specify a solution—giving space for future innovation and low-risk exploration. A starting point may be as simple as generating active discussions between industry, academia, policymakers and regulatory bodies. From this, discussions on tangible form can arise as there are many ways to address the circularity challenge.

## 9. Imagining the ideal system innovation portfolio

Designing for innovation in an environment that lacks precedents is incredibly challenging, but for industry to move beyond incremental shifts, it needs to be able to see a future operating reality when the innovation ecosystem is thriving, so this kind of constructivist activity provides a bridge between research and action [43]. The initial missions set direction, but as they mature they grow into a systemic endeavour. As Medzinski *et al.* [44] say: ‘Many innovations with a transformative impact are system innovations. System innovation is a portfolio of interdependent and mutually reinforcing innovations, which together have a potential to transform systems. The impact of system innovations depends on the strength of synergies between its elements rather than only on the disruptiveness of individual technologies’.

**Figure 5.**
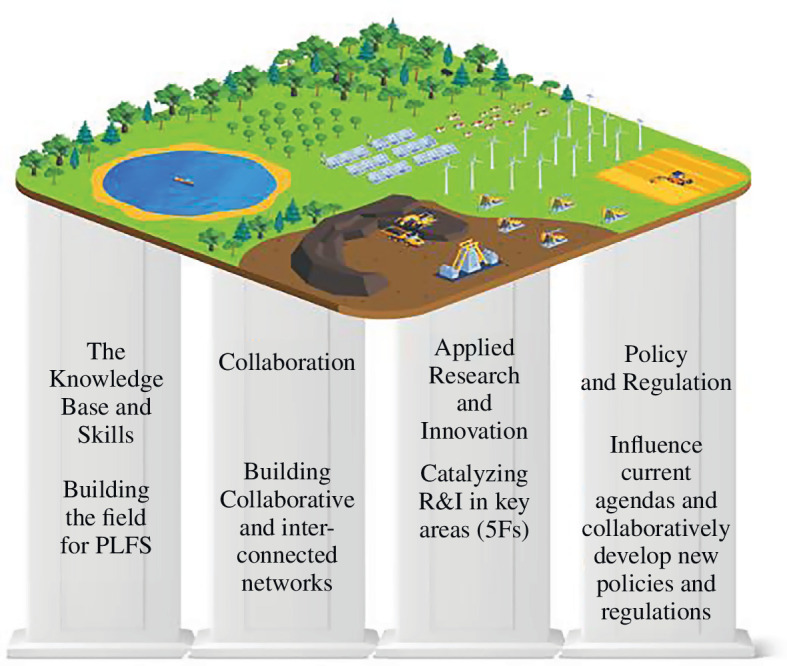
The components of a holistic portfolio.

The final Mission Lab engaged the task force to design an ideal-type innovation portfolio for PLFs [45]. This exercise concentrated on what was both desirable and technically possible, but not necessarily economically viable. This focus encourages ambition—allowing for the imagination of preferable futures and leaving their viability as a future consideration. The purpose of this portfolio visioning exercise was to highlight collective latent demand and bring forth novel ideas that reveal the common needs of industry players in a collaborative environment. The outcome was a set of speculative designs for projects that sat within a system innovation portfolio [46] with four essential pillars of change that make up a holistic approach to PLF: knowledge and skills, networks and partnerships, research and innovation programmes, engagement with policymakers, regulatory and standard-setting organizations (see figure 5).

## 10. Driving common-good innovation

The end goal of the missions was to seed game-changing research and innovation programmes, and so we invited the group to consider what was collectively possible when focusing on common-good scenarios. By asking industry representatives to think collectively about what was needed to generate mission-oriented innovation ecosystems [47] and ‘shared value’, the task force acknowledged that this process would require greater collaboration than conventional models of open innovation or pre-commercial prosperity partnerships could provide.

There are many industry rules and norms that prevent collaboration in regular corporate innovation settings, so these Mission Labs provided a unique environment for participants to contribute to a process for how common-good practices might be embraced. As Mazzucato says: ‘A market-shaping approach to the common good must change how the public and private sectors work together; there needs to be a move towards a mutualistic relationship characterized by shared goals geared towards a common goal’ [48].

**Figure 6.**
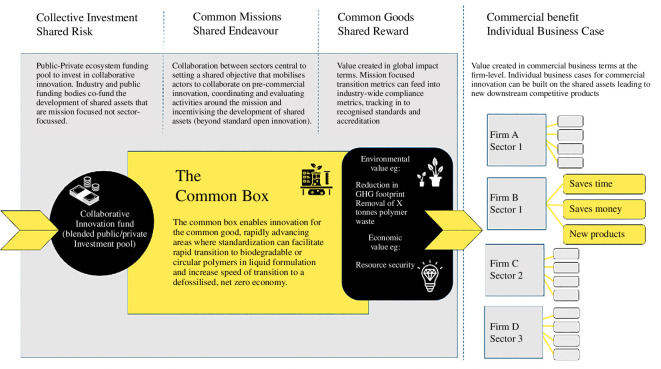
The speculative design for the evolution of open innovation.

In one co-design session, we were presented with the speculative idea of a ‘common box’—a novel approach to pre-commercial innovation in which a protected shared space could house common-interest research and innovation activities (such as life-cycle analysis and shared digital infrastructure) enabling faster transitions and cutting out duplication of effort driven by firm-level industry–academia partnerships. The common box is an extension of the concept of open innovation and if appropriately resourced and managed, this kind of common-interest innovation model could in turn provide a platform for faster and more effective firm-level innovation and value creation (see figure 6 for the speculative illustration of this developed through dialogue with Jason Harcup, Global Vice President for Skin Care Research & Development at Unilever).

## 11. Conclusions

**Figure 7.**
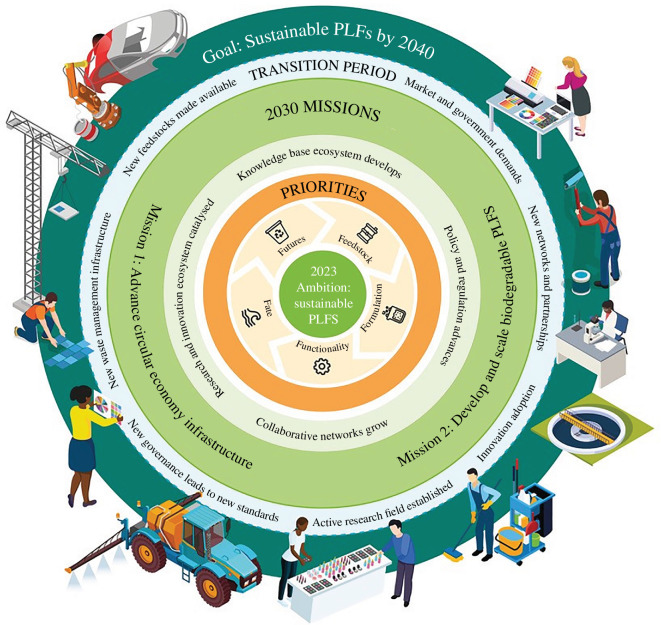
The roadmap to sustainable PLF. This roadmap was collaboratively co-designed by the industrial task force through a series of interactive mission labs.

In July 2023, the RSC released its final report with a roadmap that outlines the path to make PLFs sustainable by 2040 (see figure 7). By focusing on large-scale industry transformation rather than firm-level innovation, the scale of demand for innovation from the whole of industry was revealed. This roadmap now serves as an industry-wide strategy for sustainability for PLFs, giving clarity to what this transition may look like and has catalysed a programme of partnerships and consortia proposals to UKRI and the European Commission.

The assets developed in this process include: two critical missions for a rapid transition to sustainable PLF, a sustainability framework, a roadmap and a speculative design for the ideal R&I portfolio that defined the four key pillars of a flourishing sustainable PLF ecosystem. The roadmap provides clarity for the transition route to green carbon-based supply chains and this process offers lessons for how bottom-up contributions from industry like this could be integrated into innovation policy to support both fast and deep sustainability transitions. Future iterations will need to include ways to convene policymakers in a transparent way to inform a clear policy, standards and regulatory framework governing PLF, and ways to explore novel routes to patient finance for new infrastructure development. Some of the challenges that still lie ahead are outlined below.

### (a) Challege 1: build a ‘consequences observatory’ for the transition

Mapping the consequences for an industry-wide transition will help stakeholders to assess effects on the industry value chain, as well as test shared metrics, models and governance frameworks against adjacent sustainability models such as the ‘just transition.’ [49].

### (b) Challenge 2: experiment with economics

Moving from ‘technically feasible’ to ‘economically viable’ is the biggest leap that is somewhat obscured in the gaps between the sustainability-transition literature and the field of innovation policy. To coordinate large-scale shifts in the industry requires more than signals from academia and industry, it requires finance, scientific consensus, innovation infrastructure and regulatory and policy readiness to support the move from virgin fossil to green feedstocks. Finding the right way forward will require experimentation.

### (c) Challenge 3: foster deep and collaborative networks within the industry

The task force demonstrated that there is significant scope for more pre-competitive collaboration to speed up innovation such as sharing findings about polymers that have been tested and found to be ineffective. Equally, the industry will need to partner with academia and with research organizations that have important expertise or influence on the wider PLF field. The importance of a value-chain-wide shared life-cycle analysis was posited, emphasizing the industry-wide value of sharing LCA calculations to guide decisions for sustainability challenges, rather than burying LCA within firm-level proprietary research. Sharing knowledge through LCA as an innovation commons could help whole industry transitions, supporting sustainable practices for PLF manufacture, use, recycling and disposal as a shared endeavour.

### (d) Challenge 4: scale applied research and innovation

Our aim was to catalyse transformative innovation, which meant that developing a new R&I ecosystem was key. Since the launch of the roadmap, the RSC has engaged with funding bodies to connect PLFs to existing and emerging R&I agendas and cultivated a growing portfolio of funded projects to deliver on both missions.

### (e) Challenge 5: actively engage with policymakers

Engaging proactively to identify priorities, gaps and opportunities for innovation and the management of safer sustainable household chemicals is an ongoing and essential task. There are many challenges that lie ahead in the transition to sustainable PLFs. Without a single solution, it is important to take a portfolio approach to innovation for sustainable PLFs and recognize that there will be multiple approaches to sustainability and many decisions to be made on the chemical technologies required to manufacture PLF. Complex issues such as resource security and price stability will undoubtedly inform whether feedstocks can switch to secondary fossil- or bio-based resources [50]. Policy mandates need to remain ambitious while working within the biophysical limits of production systems. Missions may provide the policy mechanism to enable this transition.

## Data Availability

This article has no additional data.
